# Debate: Where to next for universal school‐based mental health interventions? Addressing the mental health crisis among young people in low‐ and middle‐income countries

**DOI:** 10.1111/camh.12752

**Published:** 2024-12-07

**Authors:** Bronwyne Coetzee, Maria Loades

**Affiliations:** ^1^ Department of Psychology Stellenbosch University Stellenbosch South Africa; ^2^ Department of Psychology University of Bath Bath UK

**Keywords:** Prevention, school‐based, mental health, universal prevention, low‐ and middle‐income

## Abstract

In their paper, Birrell et al. (*Child and Adolescent Mental Health*, 2025) are rightly concerned about the future of universal school‐based mental health interventions. They highlight the successes and failings of these interventions and demonstrate that there is still much to learn about their potential in preventing or mitigating the onset of common mental health conditions like anxiety, depression, trauma and substance use. But encouragingly, and thanks in part to the potential these interventions hold for addressing the mental health gap in low and middle‐income countries (LMIC), the authors advise that we as researchers need to take a step back in order to take a better step forward. They suggest that instead of abandoning ship altogether, we should critically examine the shortcomings of these interventions in their current format and work more closely with young people themselves to design and deliver interventions, which have long‐term benefits for their communities. In this paper, we want to emphasise the urgency with which these interventions, developed in this way, are needed in LMIC. We emphasise the need to co‐develop, adapt, test and evaluate school‐based interventions, and the potential they hold for reducing the burden of mental health care in resource‐constrained settings.

## Introduction

A colleague of ours recently used the metaphor of ‘*running the gauntlet*’ when explaining vulnerability to poor mental health among young people living in poor and resource‐limited settings like low and middle‐income countries (LMIC). She explained that running the gauntlet was a historical practice of corporal punishment where an individual was forced to run between two rows of people who would try to strike them as they passed, and eventually at some point or another, they would be hit. Indeed, in environments characterised by significant adversity, including poverty, violence, maltreatment, war and conflict, environmental degradation, climate change, epidemics and pandemics, at some point young people will be ‘hit’ and mental health problems will emerge, and some will be hit harder and more often than others. So, what do we do? What, where and how can support be offered to protect young people from being hit, or to ‘soften the blow’? How can we use available resources and infrastructure, and offer support equitably in places where young people already spend time? Without a doubt, in schools.

Indeed, schools provide an opportunity to reach young people where they spend considerable amounts of their time and provide a platform to raise awareness about mental health. School‐based interventions offered to all young people can reduce mental health related stigma and support positive school climates that promote and prioritise well‐being. In South Africa, more than 90% of young people (7–17 years) attend school making them ideal settings to reach young people (Hall, 2024). Yet, we know very little about the potential of schools as vehicles for providing mental health support in these settings. Similarly, we know very little about universal school‐based mental health support in school settings in LMIC (Bradshaw et al., 2021). Despite mixed evidence of their effectiveness in much better‐resourced settings, universal school‐based mental health interventions are under‐explored in LMIC, which means that there is much to be understood about their potential to provide young people with the skills they need to buffer life's, often incomprehensible, hardships. As such, the evidence from the ‘Global North’ while compelling and concerning, does not negate the importance of better understanding the potential of these interventions in LMICs where the mental health treatment gap is large, the vulnerability factors are high for all young people, and few skilled professionals are available.

However, as Birrell et al. (2025) correctly point out, there is overwhelming evidence from high income countries where these interventions have been most extensively tested, that highlight their inability to sustain the reduction of symptoms of depression and anxiety in the long term and even can have harmful effects (Birrell et al., 2025; Andrews et al., 2023; Kuyken et al., 2022). These findings are certainly concerning, as young people in LMIC do not need hits that could otherwise be avoided, but we agree that instead of abandoning the universal intervention approach altogether, that these findings offer an important opportunity to pause and think, and even take a step back to attempt to understand how to improve their design and delivery towards better outcomes.

Much less is known about universal school‐based interventions in LMIC, especially those with a focus on prevention. Nonetheless there is some good evidence that demonstrates that these interventions are both feasible and acceptable and can be relatively easily and flexibly delivered in these culturally and linguistically diverse settings. For example, the Strengthening Evidence Base on School‐Based Interventions for Promoting Adolescent Health (SEHER) program in India (Shinde et al., 2018, 2020), which Birrell et al. (2025) point out in their piece, utilised trained lay counsellors and teachers to deliver school‐based mental health support, and showed positive outcomes (when delivered by the lay counsellors) such as reduced depressive symptoms, improved school climate, and decreased bullying. These findings demonstrate the capacity of school‐based interventions to create safe and supportive learning environments, which are essential for promoting mental health. Similarly, the 4 Steps To My Future (4STMF) pilot intervention in South Africa showed that universal interventions can be delivered by trained postgraduate psychology students rather than specialised mental health professionals (i.e. a task‐sharing approach) and that the program improved self‐esteem and emotional regulation, had high engagement rates and positive feedback from young people and their teachers (Coetzee et al., 2024).

Indeed, and as shown in these two examples, a key strength of universal school‐based interventions is that they can be flexibly delivered, when tailored appropriately, by non‐specialist facilitators. This task‐shifting approach is essential in LMIC and has shown to help overcome various barriers to accessing healthcare, especially among underserved and hard to reach groups like those living in rural settings (Hoeft, Fortney, Patel, & Unützer, 2018). Moreover, universal programs mitigate the stigma often associated with targeted and indicated mental health interventions. By providing mental health interventions to all young people, these programs reduce the risk of singling out individuals based on perceived vulnerability. In our work with the 4STMF program, we observed that young people felt comfortable discussing topics related to the content of the sessions in a group setting. Such open dialogue helps to address stigma and create a positive school climate that prioritises well‐being, which in the long term may prevent common mental health conditions.

However, implementing universal school‐based mental health interventions in LMICs is not without its challenges. Large class sizes, limited resources, and competing exam schedules mean there are many factors which may compromise their delivery and evaluation. But these challenges may be overcome by collaborating with local non‐governmental organisations (NGOs) that support the psychosocial well‐being of young people, parents and teachers in resource‐limited schools, retain a permanent presence within schools and can integrate these programmes into their own scope of work. For example, the 4STMF programme benefited from the involvement of an NGO, Community Keepers, which operated within the schools within which the pilot was conducted. This collaborative approach ensured that the sustainability of the programme was kept in mind from the outset, that the programme fitted the context and had community buy‐in.

As Birrell et al. (2025) point out, one of the biggest shortcomings of universal school‐based interventions to date and particularly applicable to LMICs, is that there has been limited involvement of young people themselves to co‐inform design, delivery, what is measured and what matters. Universal interventions that are youth‐informed, co‐developed and led, or adapted in close consultation with key stakeholders in the local community, have the potential to offer wider benefits such as promoting mental health awareness and help‐seeking among caregivers. Youth engagement is a critical component of effective school‐based mental health programs and young people in LMICs themselves have reported that they are eager to participate in mental health initiatives (Pavarini et al., 2023). Young people in these settings have reported that integrating mental health education into school curricula empowers them with knowledge and skills to support themselves and their peers. In our work, young people have shared that interventions like these will equip them with skills to help their caregivers. As such, the ripple effects of such interventions need to be explored and better understood.

## Conclusion

We believe that universal school‐based mental health interventions hold much potential to address the growing mental health burden among young people in LMIC, and globally. Further, these interventions are likely to provide young people with the necessary skills needed to ward off the many hits they are exposed to. We strongly advocate for these interventions to be co‐designed with young people to not only ensure they are contextually appropriate, but that they have true relevance and meaning to young people who need to integrate the core components of these interventions into their lives. There is also much to understand and unpack about the wider benefits of these interventions, in schools, communities and beyond.

## Conflict of interest statement

The authors declare no conflict of interest.

## Author contributions

B.C. conceptualised the article, conducted searches of the literature and wrote the first and final draft of the manuscript. M.L. provided input on the initial draft and reviewed the final version.

## Funding information

M.L. (Advanced Fellowship, 302929) is funded by the National Institute for Health Research (NIHR) for this research project. The views expressed in this publication are those of the author(s) and not necessarily those of the NIHR, NHS or the UK Department of Health and Social Care.

## Ethical information

No ethical approval was required for this article.

## Data Availability

Data sharing is not applicable to this article as no new data were created or analyzed in this study.
